# Morphological characteristics of the plantar calcaneocuboid ligaments

**DOI:** 10.1186/s13047-020-00443-7

**Published:** 2021-01-07

**Authors:** Mutsuaki Edama, Tomoya Takabayashi, Hirotake Yokota, Ryo Hrabayashi, Chie Sekine, Ikuo Kageyama

**Affiliations:** 1 Institute for Human Movement and Medical Sciences, Niigata University of Health and Welfare, Shimami-cho 1398, Kita-ku, Niigata City, 950-3198 Japan; 2 Department of Anatomy, School of Life Dentistry at Niigata, Nippon Dental University, Niigata, Japan

**Keywords:** Long plantar ligament, Short plantar ligament, Calcaneocuboid joint

## Abstract

**Background:**

The aim of this study was to clarify the differences in morphological features between the long plantar ligament (LPL) and the short plantar ligament (SPL).

**Methods:**

This investigation examined 50 legs from 25 Japanese cadavers. The LPL and SPL of each leg were classified into one of three types based on the shape and number of fiber bundles. Then, fiber bundle length, fiber bundle width, and fiber bundle thickness were measured.

**Results:**

The LPL was rectangular in shape (Type I) in 12%, hourglass shape (Type II) in 62%, and triangular in shape (Type III) in 26%. The SPL was a single fiber bundle (Type I-a) in 26%, a surface fiber bundle and a deep fiber bundle (Type I-b) in 60%, and a surface fiber bundle (medial and lateral) and a deep fiber bundle (Type II) in 14%. Regarding the morphological characteristics, there were no significant differences among the types in the LPL, but there were differences between types and between surface and deep fiber bundles in the SPL.

**Conclusions:**

For the LPL, the hourglass shape is the most common type. However, there appeared to be no functional difference due to the difference in the shape of the LPL, since there were no significant differences among the types in the LPL. For the SPL, there were types of single, double and triple fiber bundles; there may be functional differences based on the number of fiber bundles and between superficial and deep fibers.

## Background

Isolated injuries involving the calcaneocuboid joint are rare and frequently overlooked. Indeed, these injuries are a cause of symptoms after an inversion injury of the foot, and the resulting clinical manifestations may mimic a lateral ankle ligament injury, which is more common [1–4]. These injuries predominantly affect young and active persons and are of significant economic importance [1, 2]. Furthermore, patients with injuries involving the calcaneocuboid joint require different treatment approaches than those with the more frequently occurring lateral ankle ligament injury [1]. With a greater understanding of the morphological characteristics of the ligament, an accurate and timely diagnosis can be made, preventing late sequelae from developing and obviating complicated surgical procedures.

The calcaneocuboid joint is formed by the quadrilateral facets of the calcaneus and cuboid bones and the capsule, which is reinforced by ligaments. The four ligaments connecting the calcaneus and cuboid are: the medial calcaneocuboid ligament, a component of the bifurcate ligament; the dorsolateral calcaneocuboid ligament; the plantar calcaneocuboid ligament, or short plantar ligament; and the long plantar ligament [5, 6]. These ligaments play a major role in supporting the medial and lateral longitudinal arches [7–9]. However, morphological characteristics and the functional roles of the plantar calcaneocuboid ligaments have not been fully considered.

Previous studies [5, 6, 10] referred to the plantar calcaneocuboid ligament that was then subdivided into the long plantar ligament (LPL) and the short plantar ligament (SPL); the SPL is also known as the plantar calcaneocuboid ligament. It is generally agreed that the LPL attaches posteriorly to the inferior surface of the calcaneus between the posterior and anterior tubercles [5]. It has been reported that the superficial fibers insert into the bases of the second to fourth metatarsals (MTs) and not the distal cuboid. Previous studies suggested that the superficial fibers insert variably to the metatarsal bone [6, 11]. One study [6] reported that, in all 59 specimens examined, the LPL had an hourglass shape, and structural variations were observed in the LPL in 20.3% (12 ft), in the form of medial twisting fibers in 11.8% (7 ft), lateral twisting fibers in 3.3% (2 ft), and additional bands in 5% (3 ft). In another study of 10 ft, the shape of the LPL was hourglass to rectangular in 100% [10]. The following morphological data have been reported: length 28.5 ± 10.5 mm [5], 44.2 ± 4.4 mm [6], 38.9 ± 2.1 (lateral) mm [10], and 61.4 ± 24.4 (medial) mm [10]; and width 10.7 ± 2.8 mm [5], 10.9 ± 2.8 mm [6], and 12.2 ± 1.1 mm [10].

The SPL passes anteromedially as a widening band from the anterior tubercle of the calcaneus to attach to the plantar surface of the cuboid posterior to the ridge for the tendon of peroneus longus, and it is often described as blending with and reinforcing the calcaneocuboid joint capsule [5, 6, 10]. Some displayed superficial and deep bands, and in 23 ft (39%) of the 59 specimens, the deep band had a distinct attachment to the calcaneus [6]. The overall shape and arrangement of the SPL was triangular in 21 ft (35%), rectangular in 34 ft (59%), and trapezoidal in 4 ft (6%). In addition, an extra band or bands were also observed in 19 ft (32%), in two of which the extra bands were also twisted [6]. In another study, every SPL had at least two bands: superficial and deep [10]. A rectangular or slightly triangular superficial band was seen in 6 ft (60%), and in the remaining 4 ft (40%), it was triangular with small separate bundles [10]. The following morphological data were reported: length 18.2 ± 4.3 mm [5], 19.4 ± 3.6 mm [6], and 21.0 ± 1.9 mm [10]; width 10.3 ± 4.4 mm [6], 11.2 ± 0.8 mm [10], and 12.2 ± 3.3 mm [5]; and thickness 4.8 ± 0.3 mm [10]. Thus, there are few morphological reports of the plantar calcaneocuboid ligaments, and there is no consensus.

Therefore, the aim of this study was to clarify the differences in morphological features between the LPL and the SPL.

## Materials and methods

### Cadavers

This investigation examined 50 legs from 25 Japanese cadavers (mean age at death, 78 ± 12 years; 28 sides from men, 22 from women; 25 right sides, 25 left sides) that had been switched to alcohol after placement in 10% formalin. None showed signs of previous major surgery around the foot or ankle or any relevant deformities, and there was no obvious degeneration in all specimens. This study was approved by the Ethics Committee of our institution (18071).

### Methods

**Fig. 1:**
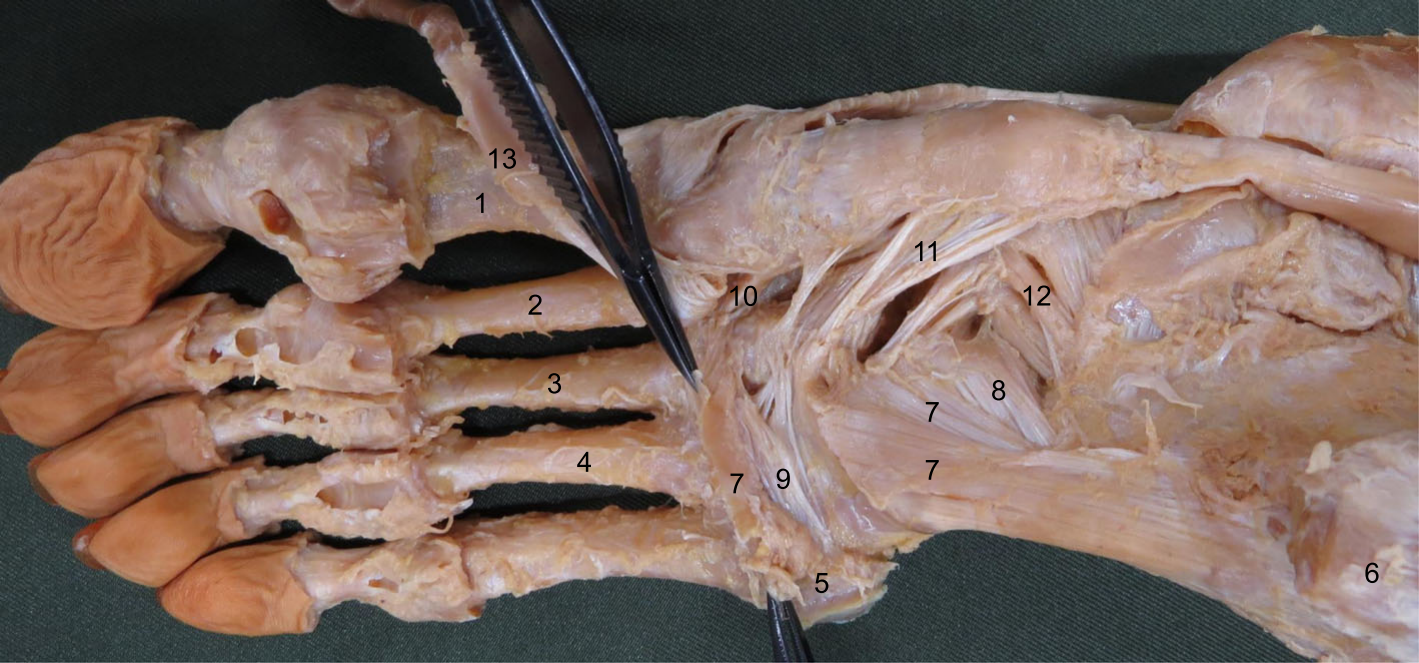
Dissection procedure for the long plantar ligament: right foot, plantar view 1: First metatarsal, 2: second metatarsal, 3: third metatarsal, 4: fourth metatarsal, 5: base of the fifth metatarsal, 6: calcaneus, 7: long plantar ligament, 8: short plantar ligament, 9: blended fiber bundle of the long plantar ligament, 10: medial cuneiform–metatarsal 2 & 3 plantar ligament, 11: posterior tibialis, 12: spring ligament (plantar calcaneonavicular ligament), 13: peroneus longus tendon

The procedures for dissection of the LPL and SPL are described below. All dissections were performed by the same examiner. Isolated specimens of the leg were created by transection 10 cm above the ankle. The skin, subcutaneous tissue, and crural fascia were removed, and the peroneus longus and brevis and the tibialis posterior were carefully dissected and inspected. The plantar aponeurosis was meticulously dissected from the flexor digitorum brevis and removed. The first, second, and third plantar layers (flexor digitorum brevis, flexor digitorum longus, flexor hallucis longus, lumbricals, plantar quadratus, abductor hallucis, abductor digiti minimi) were then dissected and excised. Upon reaching the fourth layer, the peroneus longus tendon was once again identified behind the lateral malleolus and then followed into the plantar aspect of the foot. In all specimens, the LPL formed the roof of the peroneus longus canal, blending with the canal before inserting on the metatarsal bases. To clarify the ligaments, the plantar muscles were dissected and excised (Fig. 1). The LPL and SPL of each leg were then classified into one of the three types based on the morphological characteristics and the number of fiber bundles.

Fiber bundle length, fiber bundle width, and fiber bundle thickness were measured for the LPL and the SPL. Fiber bundle length, fiber bundle width, and fiber bundle thickness were measured in the central portions of the LPL and SPL using calipers (Digital Caliper, Shinwa, Niigata, Japan). All measurements were made by the same examiner, with each site measured three times, and the mean value and standard deviation were then calculated.

### Statistical analysis

Statistical analyses were performed using SPSS (version 24.0, SPSS Japan Inc., Tokyo, Japan). The chi-squared test was used for comparisons between men and women and between right and left sides in the classifications based on differences in the type. Comparisons of fiber bundle length, fiber bundle width, and fiber bundle thickness in each type were made with an unpaired *t*-test, one-way repeated measures analysis of variance (ANOVA), and Bonferroni’s method. Comparisons of fiber bundle length, fiber bundle width, and fiber bundle thickness among types were made with a paired *t*-test. The level of significance was 5%.

## Results

### Classification of the long plantar ligament

**Fig. 2:**
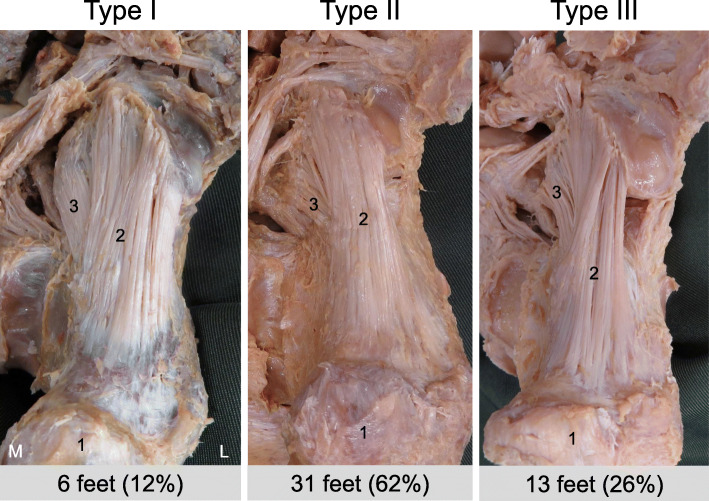
Classification of the long plantar ligament. Type I: the LPL has a rectangular shape. Type II: the LPL has an hourglass shape. Type III: the LPL has a triangular shape. 1: calcaneus, 2: long plantar ligament, 3: short plantar ligament, L: lateral, M: medial

Using the classification based on differences in the shape, there were three types: Type I, Type II, and Type III. The types were as follows: Type I, the LPL was a rectangular shape; Type II, the LPL was an hourglass shape; and Type III, the LPL was a triangular shape. Type I was seen in 6 ft (12%), Type II in 31 ft (62%), and Type III in 13 ft (26%) (Fig. 2).

There were no significant differences between males and females and between left and right sides (Table 1).

**Table 1:** Comparison of classifications of the long plantar ligament by sex and laterality

	Type I	Type II	Type III
Male (*n* = 28)	3 (6)	19 (38)	6 (12)
Female (*n* = 22)	3 (6)	12 (24)	7 (14)
Right (*n* = 25)	3 (6)	16 (32)	6 (12)
Left (*n* = 25)	3 (6)	15 (30)	7 (14)

Values are reported as number of specimens (%)

### Classification of the short plantar ligament

**Fig. 3:**
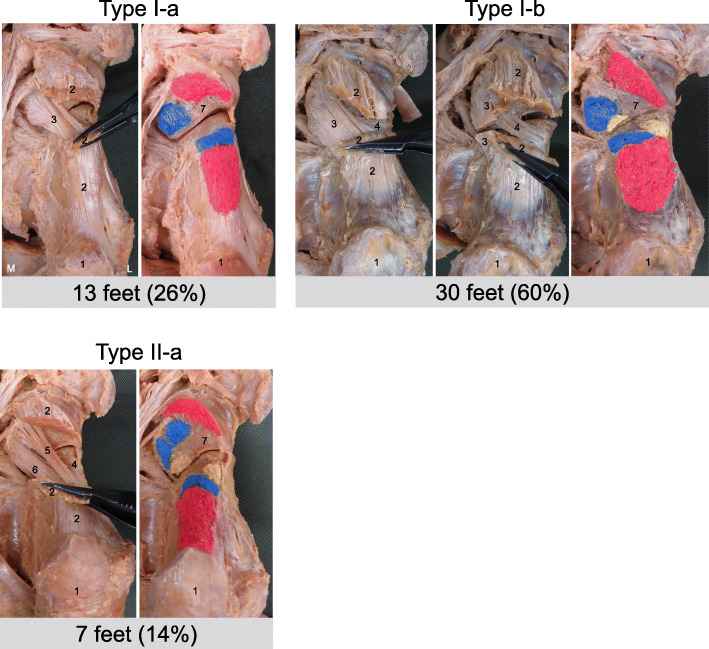
Classification of the short plantar ligament. Type I-a: the SPL is a superficial (single) fiber bundle. Type I-b: the SPL is a superficial fiber bundle and a deep fiber bundle. Type II: the SPL is a superficial fiber bundle (medial and lateral) and a deep fiber bundle. 1: calcaneus, 2: long plantar ligament, 3: the superficial fiber bundle of the short plantar ligament, 4: the deep fiber bundle of the short plantar ligament, 5: superficial fiber bundle (lateral) of the short plantar ligament, 6: superficial fiber bundle (medial) of the short plantar ligament, 7: cuboid, L: lateral, M: medial, Red area: the footprint of the long plantar ligament at the calcaneus and cuboid. Blue area: the footprint of the superficial fiber bundle of the short plantar ligament at the calcaneus and cuboid. Yellow area: the footprint of the deep fiber bundle of the short plantar ligament at the calcaneus and cuboid

Using the classification based on differences in the number of fiber bundles, there were three types: Type I-a, Type I-b, and Type II. The types were as follows: Type I-a, the SPL was a single (superficial) fiber bundle; Type I-b, the SPL was a surface fiber bundle and a deep fiber bundle; Type II, the SPL was a surface fiber bundle (medial and lateral) and a deep fiber bundle. Type I-a was seen in 13 ft (26%), Type I-b in 30 ft (60%), and Type II in 7 ft (14%) (Fig. 3).

Every SPL was rectangular in shape. The deep fiber band that reinforced the calcaneocuboid joint capsule was identified with a distinct attachment to the calcaneus and showed a more oblique orientation.

There were no significant differences between males and females and between left and right sides (Table 2).

**Table 2:** Comparison of classifications of the short plantar ligament by sex and left and right sides

	Type I-a	Type I-b	Type II
Male (*n* = 28)	8 (16)	16 (32)	4 (8)
Female (*n* = 22)	5 (10)	14 (28)	3 (6)
Right (*n* = 25)	7 (14)	14 (28)	4 (8)
Left (*n* = 25)	6 (12)	16 (32)	3 (6)

Values are reported as number of specimens (%)

### Morphological characteristics of the long plantar ligament (Table 3)

There were no significant differences among the types in the LPL.

**Table 3:** Morphological characteristics of the long plantar ligament

	Type I	Type II	Type III
Length (mm)	47.4 ± 3.8	44.3 ± 5.9	47.9 ± 5.9
Width (mm)	14.7 ± 1.8	14.2 ± 2.4	13.7 ± 2.6
Depth (mm)	1.7 ± 0.3	2.0 ± 0.5	2.1 ± 0.6

Values are means ± standard deviation

### Morphological characteristics of the short plantar ligament (Table 4)

The superficial fiber bundle was significantly wider in Type II than in Type I-a (*p* < 0.05) and Type I-b (*p* < 0.01). The superficial fiber bundle was significantly thicker in Type II than in Type I-b (*p* < 0.05).

**Table 4:** Morphological characteristics of the short plantar ligament

	Type I-a (***n*** = 13)	Type I-b (***n*** = 30)		Type II (***n*** = 7)			
	Superficial	Superficial	Deep	Superficial			Deep
				Lateral	Medial	Average/Total	
Length (mm)	21.3 ± 3.1	23.7 ± 4.8 ^d^	17.7 ± 4.6	26.3 ± 7.8	22.1 ± 3.2	26.3 ± 7.8 ^e^	18.2 ± 2.9
Width (mm)	12.3 ± 2.3	11.6 ± 2.2 ^d^	6.3 ± 1.0	6.2 ± 2.2	9.4 ± 2.7	15.6 ± 4.1^a, b, e^	5.2 ± 1.6
Depth (mm)	1.6 ± 0.7	1.5 ± 0.6 ^d^	0.8 ± 0.4	0.8 ± 0.4	1.4 ± 0.5	2.2 ± 0.6^c, e^	0.6 ± 0.3

Values are means ± standard deviation

^a^*p* < 0.05 vs. superficial fiber bundle of Type I-a, ^b^*p* < 0.01 vs. superficial fiber bundle of Type I-b

^c^*p* < 0.05 vs. deep fiber bundle of Type I-b, ^d^*p* < 0.01 vs. deep fiber bundle of Type I-b, ^e^*p* < 0.01 vs. deep fiber bundle of Type II

The fiber bundle length, width, and thickness were significantly smaller (*p* < 0.01) in the deep fiber bundles than in the superficial fiber bundles in Type I-b and Type II.

## Discussion

This study clarified the morphological characteristics of the plantar calcaneocuboid ligaments in Japanese cadavers. To the best of our knowledge, there have been no such detailed anatomical studies of these ligaments previously.

For the LPL, the hourglass shape (Type II) was the most common shape, in 62%, followed by the triangular shape (Type III) in 26% and the rectangular shape (Type I) in 12%. A previous anatomical study [6] reported that the LPL had an hourglass shape in 79.7%, and structural variations (twisting fibers and additional bands) were observed in 20.3%. In another study [10], the shape was hourglass to rectangular in 100% of the sample. Although the proportions are different, it appears certain that the hourglass shape is the most common type. However, there may be no functional differences due to the differences in the shape of LPL, since there were no significant differences among the types in the LPL.

For the SPL, in the present study, a single fiber bundle (Type I-a) was seen in 26%, and double and triple fiber bundles (Type I-b and Type II) were seen in 74%. In previous studies, the SPL had an extra band or bands in 39% [6] and 100% [10]. Although the proportions are different, there are types of single, double and triple fiber bundles. In the present study, every SPL was rectangular in shape. In previous studies, the overall shape of the SPL was triangular in 35%, rectangular in 59%, and trapezoidal in 6% [6], with a rectangular or slightly triangular superficial band in 60% and a triangular band with small separate bundles in 40% [10]. The reasons for the difference between the results of the present study and the results of previous studies are unclear and require further investigation. As for the SPL, since morphological characteristics differ between types and between surface and deep fiber bundles, there may be functional differences in the number of fiber bundles and between surficial and deep fibers.

This study has some limitations. First, the morphological characteristics of the LPL and SPL alone were investigated using fixed cadavers, and biomechanical examinations were not conducted. Second, since all of the cadavers were Japanese, it is unclear whether the present findings apply to cadavers of other ethnicities. Thus, future studies are required to evaluate variations according to ethnic origin.

## Conclusions

For the LPL, the hourglass shape was the most common type. In addition, it appears that there may be no functional differences due to the differences in the shape of the LPL, since there were no significant differences among the types in the LPL. For the SPL, there were types of single, double and triple fiber bundles, and every SPL was rectangular in shape. There may be functional differences based on the number of fiber bundles and between superficial and deep fibers. In the future, an in vivo study using ultrasound or MRI examination is needed.

## Data Availability

The data that support the findings of this study are available from the corresponding author upon reasonable request.
